# Using a theory of planned behaviour framework to explore hand hygiene beliefs at the ‘5 critical moments’ among Australian hospital-based nurses

**DOI:** 10.1186/s12913-015-0718-2

**Published:** 2015-02-13

**Authors:** Katherine M White, Nerina L Jimmieson, Patricia L Obst, Nicholas Graves, Adrian Barnett, Wendell Cockshaw, Phillip Gee, Lara Haneman, Katie Page, Megan Campbell, Elizabeth Martin, David Paterson

**Affiliations:** School of Psychology and Counselling, Queensland University of Technology, Victoria Park Road, 4059 Kelvin Grove, Australia; Institute of Health and Biomedical Innovation, Queensland University of Technology, 60 Musk Avenue, 4059 Kelvin Grove, Australia; School of Management, Queensland University of Technology, 2 George Street, 4000 Brisbane, Australia; School of Psychology, The University of Queensland, 4072 St Lucia, Australia; The University of Queensland Centre for Clinical Research, Royal Brisbane and Women’s Hospital, 4029 Herston, Australia

**Keywords:** Infection prevention, Health care-associated infections, Nurses, Attitudes, Barriers, Theory of planned behaviour

## Abstract

**Background:**

Improving hand hygiene among health care workers (HCWs) is the single most effective intervention to reduce health care associated infections in hospitals. Understanding the cognitive determinants of hand hygiene decisions for HCWs with the greatest patient contact (nurses) is essential to improve compliance. The aim of this study was to explore hospital-based nurses’ beliefs associated with performing hand hygiene guided by the World Health Organization’s (WHO) 5 critical moments. Using the belief-base framework of the Theory of Planned Behaviour, we examined attitudinal, normative, and control beliefs underpinning nurses’ decisions to perform hand hygiene according to the recently implemented national guidelines.

**Methods:**

Thematic content analysis of qualitative data from focus group discussions with hospital-based registered nurses from 5 wards across 3 hospitals in Queensland, Australia.

**Results:**

Important advantages (protection of patient and self), disadvantages (time, hand damage), referents (supportive: patients, colleagues; unsupportive: some doctors), barriers (being too busy, emergency situations), and facilitators (accessibility of sinks/products, training, reminders) were identified. There was some equivocation regarding the relative importance of hand washing following contact with patient surroundings.

**Conclusions:**

The belief base of the theory of planned behaviour provided a useful framework to explore systematically the underlying beliefs of nurses’ hand hygiene decisions according to the 5 critical moments, allowing comparisons with previous belief studies. A commitment to improve nurses’ hand hygiene practice across the 5 moments should focus on individual strategies to combat distraction from other duties, peer-based initiatives to foster a sense of shared responsibility, and management-driven solutions to tackle staffing and resource issues. Hand hygiene following touching a patient’s surroundings continues to be reported as the most neglected opportunity for compliance.

## Background

Improving hand hygiene among health care workers (HCWs) is the single most effective intervention to reduce the risk of health care associated infections (HAIs) in hospitals. As effective hand hygiene practices can mitigate the occurrence of HAIs, increased hygiene compliance, then, can help reduce the associated detrimental effects on patient health outcomes and the economic burden on health systems [1]. In 2009, the World Health Organization (WHO) adopted new global guidelines for hand hygiene that included adherence to 5 critical moments for hand hygiene for patient care that Hand Hygiene Australia [2] has worded specifically as: before touching a patient (Moment 1), before a procedure (Moment 2), after a procedure (Moment 3), after touching a patient (Moment 4), and after touching a patient’s surroundings (Moment 5). The guidelines refer to alcohol-based hand rub as the recommended method for hand hygiene when hands are not visibly soiled and that the rub should contain an emollient to protect the skin. Published hospital HCW compliance data indicate a relatively high level of safe practice although the compliance rates differed across some of the moments [3] suggesting opportunities for improvement. As nurses have the most physical contact with patients [4], it is important to understand the beliefs underlying hospital-based nurses’ hand hygiene decisions from a sound theoretical framework which can then inform intervention strategies to encourage greater compliance.

The Theory of Planned Behaviour (TPB; [5]) is a well-validated decision-making model that has been applied to hand hygiene in hospital and other contexts [6-11]. The TPB proposes that the best determinant of behaviour is intention which is influenced by three factors: attitude, subjective norm, and perceived behavioural control. Attitude refers to positive or negative evaluations of the behaviour (e.g., performing hand hygiene is good); subjective norm refers to perceptions of pressure from others to perform the behaviour (e.g., important others would want me to perform hand hygiene); and perceived behavioural control refers to perceptions of the ease or difficulty of performing the behaviour of interest (e.g., it would be easy for me to perform hand hygiene). Perceptions of control are also considered to directly influence behaviour. The TPB’s belief base proposes that attitudes are determined from the individual’s beliefs about the advantages/disadvantages of performing the behaviour (behavioural beliefs; e.g., performing hand hygiene will result in a reduction of the spread of infections). Subjective norms are determined by a person’s beliefs about whether important referents approve/disapprove of them performing the behaviour (normative beliefs; e.g., other nurses would approve of me performing hand hygiene). Perceived behavioural control is based on the individual’s beliefs about whether internal and external factors may prevent/assist in the performance of the behaviour (control beliefs; e.g., a lack of time might prevent me from performing hand hygiene [5]). The identification of beliefs can inform interventions designed to encourage behavioural performance by altering existing beliefs or exposure to new beliefs [12].

Previous non-TPB qualitative studies among HCWs have identified inaccessibility of hand hygiene resources as a key barrier to performance [13,14]. Emergencies, heavy workloads, frequent interruptions, lack of knowledge about hand hygiene practice, and skin irritations are additional barriers identified, with protection of oneself recognised as the main motivator (in addition to the protection to other HCWs and patients, and auditing), and doctors as the important referents [13]. Previous TPB qualitative research among hospital-based nurses identified other important referents, such as senior physicians, senior administrators, non-infection control nurses, and infection control nurses, noting lack of time and absence of physical contact with patients as barriers [11]. TPB quantitative research has identified hand damage, glove preference, and forgetfulness as barriers to hand hygiene [8]. It is important to establish whether the implementation of the new WHO guidelines of 5 critical moments from 2009 in Australian hospitals is associated with any changes/additions to the underlying beliefs influencing hand hygiene decisions identified in previous literature and, further, whether compliance is perceived to be equally important at the different moments. The aim of this study was, therefore, to qualitatively explore the behavioural, normative, and control beliefs related to performing hand hygiene as guided by the WHO 5 moments among hospital-based nurses to identify the modal salient beliefs, within a TPB framework, to target in a future quantitative study informed by the TPB, as well as potential interventions to encourage compliance.

## Methods

### Design

Focus group discussion data were evaluated with thematic content analysis [15]. The theory of planned behaviour and the 5 moments protocol provided a framework for the identification and coding of themes. This *a priori* framework approach has been advocated for applied policy research [16] and applied nursing research [17] which seeks to appraise existing policy and inform strategies to increase compliance. As the study sought to explore specific drivers of behaviour, it took a realist rather than phenomenological stance. Such a perspective has been recommended for studies of health service infection control [18].

### Data collection

The focus group discussions were conducted in English and occurred at the participating hospitals. Focus group discussions were facilitated by one researcher (LH; a provisionally registered organisational psychologist not connected to any of the hospitals) and audio recorded.

To guide the sessions, a semi-structured discussion guide was developed according to TPB guidelines [12]. Questions were designed to stimulate discussion about nurses’ hand hygiene beliefs. Additional probe questions were used to gain rich and detailed information [19]. The focus groups were conducted until no new ideas emerged but this goal was balanced with a desire to gauge responses across multiple hospital locations (at least 3) to ensure there were no hospital-specific nuances in hand hygiene beliefs, which we concluded that there was not.

To ensure a shared understanding about the current guidelines, participants were provided with a Hand Hygiene Australia [2] handout comprised of a visual and written description of the 5 moments. This information was reiterated verbally. The questions elicited information about behavioural beliefs for the advantages and disadvantages; normative beliefs about those who would approve and disapprove; and control beliefs comprising barriers and facilitators related to performing hand hygiene (see Table 1). Finally, given the recent introduction of the 5 moments for hand hygiene, and data to suggest varied levels of compliance for each moment, participants were asked “When you think about the 5 different moments of performing hand hygiene, do you think they are all equally important or are there some moments where it is more important to be performing hand hygiene than others?”

**Table 1:** **Focus group discussion guide**

**TPB component**	**Elicited beliefs**	**Question**
Behavioural beliefs	Advantages	“What are the advantages of performing hand hygiene?”
	Disadvantages	“What are the disadvantages of performing hand hygiene?”
Normative beliefs	Normative approval	“Who are the people (or groups of people) important to you who would approve of you performing hand hygiene?”
	Normative disapproval	“Who are the people (or groups of people) important to you who would disapprove of you performing hand hygiene?”
Control beliefs	Barriers	“What prevents or make it difficult for you to perform hand hygiene?”
	Facilitators	“What helps or motivates you to perform hand hygiene?”

### Ethical considerations

Participation was voluntary. Written consent was obtained following the provision of written information about the study. The research was undertaken in the city of Brisbane in Queensland, Australia with ethical approval obtained from the Queensland Health Human Research Ethics Committee (HREC/10/QPAH/180).

### Data analysis

Data gathering and analysis occurred in two key phases with distinct members of the research team participating in each phase. Group discussions were transcribed in full and verbatim by a professional transcriber not connected to the hospitals and who signed a confidentiality agreement. In the first phase, in addition to the researcher serving as the facilitator (LH), two researchers familiar with the subject matter (MC and PG) attended the focus group sessions to take notes after each focus group and confirm or clarify details where necessary, such as confirmation of the meaning of acronyms, and detail around specific procedures. In addition to seeking similarities across individuals and groups, when the facilitator identified negative or atypical cases in a focus group, she then checked these responses with later focus group participants to verify whether a view was commonly held. The transcript from each discussion was scrutinised independently by the three researchers (LH, MC, and PG) who attended the focus groups to confirm accuracy prior to analysis. This initial auditing of the data contributed to the dependability of the data in the final transcripts.

The purpose of the data analysis was to capture key, higher order themes as opposed to seeking deeper meaning (e.g., Interpretative Phenomenological Analysis [20]) or engage in theory development (e.g., Grounded Theory [21]). Hence, thematic analysis was an appropriate approach for the present study. The focus groups transcripts were analysed using an iterative process. Initial coding was carried out by an independent researcher (PO) who did not attend the focus groups and who had not been privy to the initial design of the study. The decision to approach analysis in this way was an attempt to remove any potential preconceptions that may have been held by members of the research team to increase the objectivity of the coding process. First, broad descriptive categories were identified and coded for each of the TPB belief components [22,23]. Next, coding was based on consideration of similarities and differences and relationships between categories and, therefore, refined into themes. Analysis considered not only frequency with which something was raised but also the extent to which participants in the group had elaborated or extended upon the issue. This process continued such that data were coded and recoded to accommodate new and emerging themes until no new themes resulted [24,25]. To enhance the credibility of this iterative process, several research team meetings overseen by two experienced researchers (KW and NJ) were held to scrutinise the results of coding decisions and reflect upon themes and patterns emerging in this process. Due to the practical and specific nature of the content (i.e., a structured discussion guide producing responses about a predominantly unemotional, pragmatic topic that could be fairly easily classified into the predetermined TPB belief constructs), there was little disparity, and consensus between the researchers was obtained relatively quickly and with limited discussion required.

## Results

### Participants

Five focus groups, each lasting approximately an hour in duration, were conducted in three large urban public teaching hospitals, with three sessions in the hospital with the greatest number of participants. The participants were nurses without specific Infection Control training who were currently working in Intensive Care Units (ICUs), general medicine, or general surgical wards (wards chosen by Infection Control personnel from the participating hospitals as medium or high HAI risk wards). Participants were recruited via noticeboard flyers and offered an $AUD50 shopping voucher as reimbursement for their time. Focus group times were organised around shifts to facilitate attendance. The sample (N = 27; 23 females, 4 males) of nurses participated in focus groups ranging in size from 2 to 10 participants. Participants were aged between 22 to 49 years (*Mdn* = 32 years) and ranged in nursing experience from 3 months to 23 years (*Mdn* = 5 years). The participants were: six clinical nurses, 17 registered nurses, three enrolled nurses, and one assistant in nursing (with these classifications based on increasing levels of qualifications and experience, with a clinical nurse being most senior, followed by registered nurse, then enrolled nurse, and assistant in nursing). Fifteen of the nurses worked in general medical wards, seven in surgical wards, and five in ICUs. Please see Table 2 for a description of each focus group and its participants.

**Table 2:** **Descriptive data of focus group participants (N =27)**

**Focus group number**	**n**	**Gender**	**Age**	**Length of service**	**Role type**	**Work area**	**Work status**
FG1	6	2 Males	(25-45 yrs)	(0.5-23 yrs)	2 Clinical nurses	6 General medicine	4 Full-time
		4 Females	Mdn = 30.5 yrs	Mdn = 10.5 yrs	3 Registered nurses		2 Part-time
					1 Assistant in nursing		
FG2	6	6 Females	(32-49 yrs)	(2-12 yrs)	2 Clinical nurses	4 General medicine	5 Full-time
			Mdn = 38 yrs	Mdn = 5.75 yrs	1 Enrolled nurse	2 General surgical	1 Part-time
					3 Registered nurses		
FG3	2	2 Females	(29-43 yrs)	(8-21 yrs)	1 Clinical nurse	2 Intensive care unit	2 Part-time
			Mdn = 36 yrs	Mdn = 14.5 yrs	1 Registered nurse		
FG4	10	8 Females	(22-43 yrs)	(2-20 yrs)	1 Clinical nurse	4 General medicine	8 Full-time
		2 Males	Mdn = 29 yrs	Mdn = 4.5 yrs	9 Registered nurses	3 General surgical	2 Part-Time
						3 Intensive care unit	
FG5	3	3 Females	(24-28 yrs)	(0.5-5 yrs)	2 Enrolled nurses	1 General medicine	1 Full-time
			Mdn = 24 yrs	Mdn = 4.5 yrs	1 Registered nurse	2 General surgical	2 Part-time
Total	27	23 Females	(22-49 yrs)	(0.5-23 yrs)	6 Clinical nurses	15 General medicine	18 Full-time
		4 Males	Mdn = 32 yrs	Mdn = 5 yrs	3 Enrolled nurses	7 General surgical	9 Part-time
					17 Registered nurses	5 Intensive care unit	
					1 Assistant in nursing		

The results were consistent across the group discussions and are organized around the three main topic areas that were used to frame the discussion. Table 3 provides a summary of the key concepts, themes (including the number of times a theme was expressed), and example quotations for the TPB beliefs, with salient themes noted based on the number of times a theme was mentioned (irrespective of whether a theme was raised multiple times by the same participant as transcripts did not identify quotes by each individual speaker).

**Table 3:** **Key Concepts, Themes (including number of times theme expressed), and Example Quotations of Beliefs across the Full Sample (N =27) identified by Focus Group Number (FG 1–5)**

**Concept**	**Key themes**	**Example quotations**
**Behavioural Beliefs –Advantages**	Patient protection	“Yeah, our poor patients who can pick up an infection at the drop of a hat and we're the ones walking in with a cold and stepping over them and touching them.” (FG2)
	(n = 11)	
	Self protection	“If more people think selfishly and it's like ‘I have touched that patient and I don't want their germs,’ then you will wash your hands more often. Because you don't know what your patients have got.” (FG2)
	(n = 8)	
	Infection control	“Because people are sick in hospital, you tend to think of the individual being germy; where it's not the actual individual that is germy. We all have different bacteria that live on our skins. If you touch something of somebody's, even if it's just their handbag which they touch every day, and then you sort of plonk it back down and move on to something else, the bacteria that would normally reside on that individual's skin, it could potentially move to another.” (FG2)
	(n = 7)	
**Behavioural Beliefs -Disadvantages**	Hand damage	“You don't realise until the end of the day how many times you have washed your hands and how sore and cracked they end up.” (FG2)
	(n = 9)	
	Time	“If you are busy, it adds quite an extra bit of time onto what you are actually doing.” (FG3)
	(n = 5)	
**Normative Beliefs -Supportive**	Colleagues	“I actually have seen people say, ‘Can you wash your hands?’, or something like that. So I have actually heard that question being asked of colleagues or, ‘Can you wash your hands and come give me a hand?’” (FG2)
	(n = 17)	
	Supervisors	“And because our professor, he's the senior person and the head of the unit, everyone abides by it… because he's enforcing it so diligently.” (FG4)
	(n = 7)	
	Patients	“Seems like the patients approve, that they appreciate it.” (FG2)
	(n = 6)	
	Infection control staff	“Well, yeah, they [infection control] are good. They sort of lead by example as well, but they are not sort of on your back all the time or anything. But we did know when they were doing the audit. They were walking around, looking.” (FG5)
	(n = 4)	
	Family	“Our family members, so we are not taking it back to them.” (FG1)
	(n = 2)	
**Normative Beliefs -Unsupportive**	Doctors/consultants	“There's even doctors who challenge the fact that hand washing actually prevents - like, they ask you, “Oh, where’s the study that proves it?” (FG1)
	(n = 10)	
	Patients	“…they get really offended. If you have just gone and touched them on the shoulder and you just go and wash your hands, they are like, “I don’t have any germs. Rah, rah, rah, I had a shower.”” (FG1)
	(n = 2)	
**Control Beliefs -Facilitators**	Availability of sinks/hygiene products	“Yes, with everything that we have put into place, like they are mounted on the outside of rooms just as you leave the wards, outside of every room, then you have got pumps at the end of the bed plus your wash basin in every room.” (FG2)
	(n = 21)	
	Education/training programs	“I just remember being a student in my crew, what they did, they broke us into sections and got some of us to do a 30-second wash, some of us a surgical scrub and then we all touched agar plates and then three days later had a look at our growths and I think that was a really good thing to just make it really real and show us how many bugs we could be carrying.” (FG1)
	(n = 17)	
	Infection outbreak/infectious patients	“When we get MRSA [Methicillin-resistant *Staphylococcus aureus*] or VRE [Vancomycin-resistant Enterococci], we always have a meeting and the manager re-enforces everything and tries to make sure that there's soap in every - at every sink and stuff like that. So they really up the infection control.” (FG1)
	(n = 13)	
	Auditing/infection control unit	“On a hospital level, they do a lot. They have got auditors on each ward. There's at least some staff - there will be at least two staff members that you can go to and ask questions, if you need to ask questions about hand washing. There’s knowledgeable people everywhere about infection control.” (FG5)
	(n = 11)	
	Verbal/visual reminders	“I guess putting up signs as well saying ‘wash your hands.’ I mean, just that picture.” (FG4)
	(n = 7)	
	Access to dermatologist	“They (nurse unit managers) keep on reminding us, ‘There's available help, just in case you need that cream/medication, we can help you to repair with the dermatologist.’” (FG1)
	(n = 4)	
**Control Beliefs - Barriers**	Emergencies	“If someone falls then you are not going to walk to the sink first… I guess you might forget to in that situation because your focus is basically off your hand hygiene and it's more on the patient.” (FG1)
	(n = 7)	
	Skin irritations	“I remember once on night shift I washed my hands so much that they were just - they were almost irritated from me washing them and all I had was alco wipe stuff and the sting - it was horrible.” (FG4)
	(n = 7)	
	Product/sinks not readily available	“Sometimes certain areas they are not - especially in the long-term facilities, it's hard to get to the sink sometimes because not every patient’s site has got a sink or alcohol rub.” (FG1)
	(n = 7)	
	Lack of education	“I guess you don't think of yourself as having germs either”. (FG4)
	(n = 7)	
	Distraction/forgetting	“The thing is I guess, a lot of times, you are not even aware that you have forgotten. Especially when you enter an area you might have forgotten to wash your hands and then you don't. I wouldn't know unless somebody is watching me and tells me, ‘Well, you just haven't washed your hands now.’” (FG1)
	(n = 6)	
	Lack of time/too busy	“Or that someone else has come in and opened your curtain and you are in the midst of a wash/turn, and then you have to go and close the curtains and then again, you don't have time to wash your hands - take your gloves off and wash your hands and do that.” (FG3)
	(n = 5)	
	Practical constraints	“Because it's impractical to ask the wardsman to hold your patient up, particularly if they weigh upwards to 180/200 kilos while you go and wash your hands and put on a fresh set of gloves.” (FG3)
	(n = 5)	
	Sensor taps/wasting water	“That people will turn it off and wash their hands, they are only sort of half clean but then they are unwilling to start them again because they run for too long/too little and there's thoughts of wasting water.” (FG2)
	(n = 3)	

### Behavioural beliefs: advantages and disadvantages

The nurses nominated patient protection as the most salient advantage of performing hand hygiene. Self protection and infection control also were commonly recognised by nurses as major advantages of performing hand hygiene. For the nominated disadvantages, the most salient themes were hand damage and the time taken to perform hand hygiene.

### Normative beliefs: important referents

The nurses considered work colleagues as the most salient referents supportive of their performing hand hygiene. Other supportive referents were supervisors, patients, and representatives from Infection Control. Family members of the nurses were acknowledged also given the potential for spreading infection from the workplace to home. Participants identified some doctors as unsupportive of their performing hand hygiene. Patients were cited as a group who may both approve and disapprove (given the implications of patient lack of cleanliness).

### Control beliefs: facilitating and inhibiting factors

The most frequently reported factors facilitating hand hygiene performance included the availability of sinks/hygiene products. Participants also noted that relevant education, training, and programs encouraged hand hygiene. The 5 moments campaign specifically, as readily available guidelines serving as a prompt and reminder, was considered a motivator to encourage hand hygiene. Having experienced an infection outbreak or working with infectious patients were cited also, as was auditing and the presence of infection control personnel. Participants noted that verbal and visual reminders assisted in performing hand hygiene, as would access to a dermatologist.

Emergencies were discussed by the nurses as barriers to performing hand hygiene. Other frequently nominated barriers were skin irritations, lack of availability of hand hygiene products/sinks, and a lack of education and understanding about infection control. Distraction and forgetting also were mentioned as barriers, along with lack of time and being too busy with other tasks. Practical constraints (i.e., being physically unable to interrupt some tasks) also deterred performing hand hygiene. Some participants also mentioned the issue of wasting water, especially with the need to re-trigger sensor taps to ensure a sufficient amount of water to complete hand hygiene procedures.

### Relative importance of the 5 moments of hand hygiene

When asked if one or more of the identified ‘moments’ were more critical for performing hand hygiene, participants offered mixed views. Some participants viewed all 5 moments as equally important:

‘Well, let’s be honest, there’s probably a hell of a lot more bacteria just after the exposure risk, when there’s potentially a whole lot of body fluid there. But, I mean, realistically, it’s probably just as risky with any of them’.

Other participants believed that hand hygiene was more important before and after a procedure (moments 2 and 3):

‘I think it’s probably - I don't know if it’s a fact or not - but before a procedure I would think it’s more important to wash your hands, or after a procedure, than entering a room and just touching the bed, but at the end of the day - it’s still - you have still got to move germs around if you don't wash your hands. But if I had to choose between the both, I would probably put the procedure first’.

Some participants believed that moment 5 (after touching a patient’s surroundings), while not necessarily less important for performing hand hygiene, would be more likely to be overlooked given that the perception of potential infection is less obvious (in the absence of patient contact or a current procedure) and that workers are often distracted by other tasks at this point:

‘I think they are all pretty much of a muchness. I do think that number 5 will be the one that will get missed, most likely to be missed usually and usually because of distraction, I would say; because you will be doing something and you will get either called away or something will be requested and because you haven't actually physically touched the patient, it's not in your head that you have to wash your hands’.

However, other participants discussed that, after touching a patient’s surroundings, it was fairly routine to wash one’s hands if the physical location of the sinks was near the door entry or if the patient had known infections.

## Discussion

This study explored the underlying beliefs that inform hospital-based nurses’ decisions to engage in hand hygiene, accounting for the shift in the WHO guidelines in 2009 to the 5 moments model. Nurses clearly identified the benefits of performing hand hygiene to their patients, themselves, and to hospital infection control. In contrast to previous research, protection of self was not the main advantage noted [13]. Nurses did not perceive many disadvantages to performing hand hygiene; however, damage to hands and the time required to perform hand hygiene at all 5 moments were identified as the main disadvantages.

A range of people (e.g., colleagues, supervisors, patients) were identified as sources of support for performing hand hygiene, consistent with other nurse focus group discussions [11]. However, the present study found that colleagues were the main group reported as supporting hand hygiene performance. Patients also were identified as key supportive referents, complementing recent research highlighting their potentially persuasive role in encouraging greater hand hygiene compliance among HCWs via an “It’s ok to ask” attitude [9]. For the important referents not supportive of hand hygiene, the strongest theme to emerge were reports of non-compliance and active discouragement of hand hygiene from some doctors, reported also in previous research [13]. Extending previous research and recommendations, hospital-led initiatives that empower nurses to adopt an “It’s ok to ask” attitude in relation to doctors may be an avenue for further exploration [9,13].

Nurses were able to clearly describe the key factors that facilitated hand hygiene, particularly noting that having readily accessible hand hygiene products is essential in busy working environments. Similar findings have been reported for hospital infection control initiatives in general [26]. Another major theme to emerge was the importance of education and training programs. Programs such as the 5 moments campaign prompted nurses to remember the important times to wash or clean their hands. As reported in other focus group research of HCWs, auditing of hand hygiene also was cited as a motivator and reminder to perform hand hygiene [13]. Although not raised in previous TPB studies examining hand hygiene beliefs, other reminders, such as verbal reminders from supervisors and colleagues or visual reminders from posters or signs, were cited as motivators to perform hand hygiene in the present study. Furthermore, the usefulness of the infection control unit was a theme to emerge as a helpful source for obtaining information about hand hygiene. Finally, outbreaks of infection or working with infectious patients were motivators for nurses to engage in more vigilant hand hygiene.

Congruent with the identified facilitators, the main barrier to performing hand hygiene was the non-accessibility of sinks and hand hygiene products, which is a common finding in previous focus group research [13,14]. Interestingly, in the present study, the issue of sensor taps was cited a number of times as a barrier due to concerns about wasting water, suggesting that a motivation to comply with safety standards is in conflict with environmental values. The other major themes cited as barriers involved being too busy, being distracted or forgetful, and dealing with emergency situations, consistent with other TPB belief-based research [8,11,14]. These comments highlight the disparity between nurses’ motivations to perform hand hygiene and the realities of working on a busy ward. As in previous studies, the issue of skin irritation from the use of alcohol-based rubs was raised as an obstacle to performing hand hygiene [8,13].

Given the shift to the 5 moments guidelines in Australian hospitals, it is noteworthy that there were mixed responses as to whether some of the moments were seen as more important to perform hand hygiene. While many participants recognised that infections could spread by lax practices at any of the identified moments, there was some support for the notion that the more seemingly obvious moments for infection around clinical procedures were more vital to engage in hand hygiene, and that after contact with a patient’s surroundings was the most likely scenario when performing hand hygiene would be forgotten. These results are consistent with other qualitative research findings, albeit not within a 5 moments model, where the absence of physical contact with a patient has been perceived as a barrier to hand hygiene [11] or there have been mixed views about the importance of the patient environment in hand hygiene practices [13].

When considering that the benefits of hand hygiene were recognised across all major stakeholders, including patients, staff members, and the broader hospital community, efforts to increase nurses’ compliance with guidelines should not be restricted to highlighting the benefits to any one group. The major costs associated with hand hygiene (hand damage and time) point to systemic workplace resourcing issues. The provision of appropriate hand hygiene products to prevent damage, treatment options to prevent relapses of damage (e.g., access to dermatological advice), and management of staffing demands to circumvent lower staff-patient ratios that impact on employees’ time to perform hand hygiene may help mitigate some of these costs. For the key referents, efforts should be made to foster a sense of shared responsibility for avoiding infection along the lines of safety as everybody’s business, including empowering patients and HCWs to query non-performance.

The barriers of product and sink unavailability and lack of access to adequate training highlight infrastructure and resourcing issues that can be addressed by hospital managers. Forgetting and distraction may be overcome by reiterating reminders to perform hand hygiene via visual cues in key locations (including regularly introducing new posters to attract attention). Further innovative steps to raise the profile of the 5 moments message could include initiatives such as written reminders on identification lanyards, pay notices, and official workplace email signatures. To acknowledge those who do remember, participants in the present study noted that recognition, even in the form of small tokens of appreciation, such as cups and water bottles with hygiene slogans, served as a positive reinforcement (and most likely a further reminder) to perform hand hygiene. For the 5 moments education programs, it may be useful to highlight the necessity of all 5 moments, especially for the infection risk due to forgetfulness after touching a patient’s surroundings.

### Limitations of the study

Despite the use of open-ended questions, the topics were predetermined by the TPB belief framework which may have limited the study’s scope. In addition, individual interviews may have elicited different responses to the focus groups, particularly if participants were concerned about discussing non-compliant safety behaviour within a group setting among fellow employees. The focus group discussion guide prompts also did not elicit underlying beliefs differentiated by each ‘moment’ nor were responses delineated based on their relevance to hand washing as opposed to hand rubbing which may be useful to explore in the future. Trustworthiness could have been enhanced by triangulation (gathering data from other sources, including nurse unit managers). Further, in relation to transferability, the participants were all from large public teaching hospitals in an urban centre; different beliefs may exist among nurses in smaller, private or regional hospitals. Although nurses often engage in the greatest amount of patient contact, it also should be established if similar beliefs are held by other HCWs or if belief differences exist based on specific nursing role/ward/hospital.

## Conclusion

In summary, the results of this study are concordant with, but extend upon previous research. Efforts to increase compliance should comprise individual strategies tackling prioritisation of hand hygiene among competing tasks, peer-based initiatives to cultivate adherence norms, as well as more systemic, organisational-level factors encompassing personnel and resources. Of the 5 moments, particular attention should be directed towards hand hygiene after touching a patient’s surroundings by highlighting that the opportunity for the spread of infection includes the less intrinsically apparent source of a patient’s environment. Overall, the theory of planned behaviour was useful in eliciting these beliefs and in providing practical insights to inform policy and practice encouraging greater adherence to the 5 moment initiative.

## Abbreviations

HAI/s: Health care associated infection/s
HCW/s: Health care worker/s
ICU/s: Intensive Care Unit/s
TPB: Theory of Planned Behaviour
WHO: World Health Organization
